# Acute Oroantral Communication Closure: Resorbable Collagen Membrane vs. Buccal Advancement Flap Outcomes: A Clinical Trial

**DOI:** 10.3390/jfb17030150

**Published:** 2026-03-18

**Authors:** Agnieszka Balicz, Agnieszka Szurko, Magdalena Jędzierowska, Agnieszka Kiełboń, Sylwia Wójcik, Jakub Adamczyk, Martin Starosta, Jakub Fiegler-Rudol, Tadeusz Morawiec

**Affiliations:** 1 Department of Dental Surgery, Faculty of Medical Sciences in Zabrze, Medical University of Silesia, 40-055 Katowice, Poland; swojcik@sum.edu.pl (S.W.); jadamczyk@sum.edu.pl (J.A.); tmorawiec@sum.edu.pl (T.M.); 2 Faculty of Science and Technology, University of Silesia, 40-007 Katowice, Poland; agnieszka.szurko@us.edu.pl (A.S.); magdalena.jedzierowska@us.edu.pl (M.J.); agnieszka.kielbon@us.edu.pl (A.K.); 3 Department of Dentistry, Faculty of Medicine, University of Ostrava, Syllabova 19, 703 00 Ostrava-Jih, Czech Republic; martin.starosta@osu.cz; 4 Faculty of Medical Sciences in Zabrze, Medical University of Silesia, 40-055 Katowice, Poland; jakub.fieglerrudol@gmail.com

**Keywords:** oroantral fistula, collagen, absorbable implants, surgical flaps, guided tissue regeneration, periodontal, cone-beam computed tomography, postoperative pain, oral surgical procedures

## Abstract

**Background**: Oroantral communication (OAC) is a frequent complication after the extraction of maxillary posterior teeth and requires immediate closure to prevent sinus pathology and long-term functional impairment. **Objectives**: This study aimed to compare the clinical and radiographic outcomes of acute OAC closure using resorbable heterogeneous collagen membranes with those of the conventional buccal advancement flap (Rehrmann method). **Methods**: Twenty-four patients with OACs diagnosed within 24 h post-extraction were enrolled, and 20 completed follow-up. Patients were allocated to a membrane group treated with a resorbable collagen membrane (Creos Xenoprotect) or a control group treated with a buccal advancement flap. Clinical parameters, including vestibular depth, width of keratinized gingiva, alveolar socket dimensions, postoperative complications, and pain intensity assessed using the Visual Analogue Scale, were evaluated at 1, 7, 14, and 90 days. Radiographic outcomes were assessed using cone-beam computed tomography with linear measurements and normalized bone density analysis in Hounsfield Units at baseline and 90 days. **Results**: The membrane technique provided significantly better preservation of vestibular depth, keratinized gingiva width, and alveolar socket dimensions, with significantly lower postoperative pain and fewer complications compared with the buccal advancement flap. Higher normalized bone density values were observed in the membrane group, although differences were not statistically significant. **Conclusions**: Resorbable collagen membranes represent a safe, minimally invasive, and clinically effective alternative to buccal advancement flaps for acute OAC closure.

## 1. Introduction

Oroantral communication (OAC) is a pathological connection between the oral cavity and the maxillary sinus that most frequently occurs after the extraction of posterior maxillary teeth. Because of the close anatomical relationship between molar and premolar root apices and the sinus floor, disruption of the Schneiderian membrane may occur immediately following extraction, particularly when only a thin bony lamella separates the socket from the sinus cavity [1,2,3,4]. If not promptly recognized and managed, OAC may lead to maxillary sinus contamination, chronic infection, and the development of an oroantral fistula, resulting in long-term functional impairment and the need for secondary surgical intervention [1,2,3,4]. Diagnosis is primarily clinical and may include direct probing of the extraction site, the Valsalva maneuver, or observation of fluid passage between the oral and nasal cavities. Immediate closure is generally recommended, as persistence beyond 24–48 h increases the likelihood of epithelialization and fistula formation, thereby raising the risk of chronic maxillary sinusitis [3,4]. Therefore, acute management of OAC remains an important challenge in oral surgery. The buccal advancement flap described by Rehrmann is the most applied technique for closure of acute OACs due to its simplicity and high success rate [5,6,7,8,9,10]. However, this method is associated with clinically relevant morbidity, including postoperative pain, swelling, reduction in vestibular depth, displacement of the mucogingival junction, and loss of keratinized gingiva. These sequelae may complicate oral hygiene maintenance and compromise future prosthetic or implant rehabilitation [11,12,13,14,15,16]. Alternative surgical approaches have been proposed, including the buccal fat pad flap and platelet-derived preparations, but each technique carries specific limitations related to invasiveness, technical demands, or additional procedural requirements [17,18]. Resorbable collagen membranes are widely used in guided tissue and bone regeneration and have been suggested as minimally invasive biomaterial-based options for OAC closure. These membranes provide biocompatible barrier function, support clot stabilization, and may facilitate soft tissue healing while preserving local anatomy. Heterologous collagen membranes such as Creos Xenoprotect have favorable handling properties, integrate with surrounding tissues, and resorb without the need for secondary removal, making them potentially suitable for acute post-extraction defects [19]. Nevertheless, comparative clinical evidence regarding membrane-based closure as a stand-alone approach in acute OAC management remains limited.

Therefore, the aim of this clinical study was to compare clinical and radiographic outcomes of acute OAC closure using a resorbable heterogeneous collagen membrane (Creos Xenoprotect) versus the conventional Rehrmann buccal advancement flap technique. Outcomes included vestibular depth, keratinized gingiva width, alveolar socket dimensions, postoperative complications, patient-reported pain assessed by the Visual Analogue Scale, and early radiographic bone healing evaluated using cone-beam computed tomography over a 90-day follow-up period.

## 2. Materials and Methods

### 2.1. Study Population and Group Allocation

Twenty-four patients were qualified for the study, among whom an oroantral communication was confirmed after tooth extraction. A full panel of tests was performed on 20 individuals. The patients were divided into two groups: the experimental group, which used membranes, and the control group, which used the traditional method.

This study was designed as a prospective, non-randomized comparative clinical investigation and registered under the number NCT07476664. This study was approved by the Institutional Review Board of Silesian Medical University with the code PCN/0022/KB1/94/20/21, with the approval granted on 12 January 2021. Consecutive adult patients diagnosed with an acute oroantral communication within 24 h following the extraction of a posterior maxillary tooth were screened for eligibility and enrolled. Group allocation was performed at the time of diagnosis according to a predefined treatment protocol based on the availability of the collagen membrane technique in the clinical setting. Patients treated with resorbable collagen membrane closure constituted the experimental group, whereas patients managed with the conventional Rehrmann buccal advancement flap constituted the control group. No formal randomization or allocation concealment was applied.

### 2.2. Oroantral Communication Size Assessment

The size of the oroantral communication was evaluated immediately after extraction at the time of diagnosis. The maximal diameter of the communication was measured clinically at the socket level using a calibrated periodontal probe. In all included cases, defect dimensions were additionally verified on the immediate postoperative limited-field CBCT scan performed on day 0.

### 2.3. Ethical Approval

This study was approved by the Institutional Review Board of Silesian Medical University with the code PCN/0022/KB1/94/20/2. The ethical considerations included the protection of patients’ privacy, voluntary participation with informed written consent, and the assurance of minimal harm during the study procedures. All participants were informed about the study’s purpose, potential risks, and benefits, and they had the right to withdraw from the study at any time without consequence.

### 2.4. Inclusion and Exclusion Criteria

Table 1 presents the selection criteria.

Oroantral communication size was assessed immediately after extraction at the time of diagnosis by measuring the maximal diameter of the communication using a calibrated periodontal probe and was confirmed on the immediate postoperative limited-field CBCT scan. The mean OAC diameter in the included patients was 3.5 ± 1.0 mm (range: 2–5 mm).

### 2.5. Preoperative Medical Assessment

Medical history serves to identify patients who have a higher risk of developing complications during or after closure of OAC. Cardiovascular disease, diabetes, renal dysfunction, and hematological disorders may increase the risk of complications such as bleeding, infections, and delayed tissue healing [6].

### 2.6. Surgical Procedure: Experimental Group

All surgical procedures in both groups were performed by the same experienced oral surgeon (A.B.) to minimize inter-operator variability. Postoperative clinical assessments (vestibular depth, keratinized gingiva width, and socket dimensions) were recorded by an independent examiner (T.M.) who was not involved in the surgical interventions. Prior to study initiation, the examiner underwent calibration using repeated measurements in a subset of patients to ensure measurement consistency. Radiographic measurements and region-of-interest density analyses were performed by two trained investigators, and disagreements were resolved by consensus.

Postoperative systemic antibiotics were prescribed in both groups as short-term prophylaxis due to the direct communication between the oral cavity and the maxillary sinus, which may increase the risk of sinus contamination and acute sinusitis during early healing. This approach is commonly reported in clinical management protocols for acute oroantral communications and fistulas [2,3,17]. Antibiotic regimens were standardized across groups to minimize confounding.

In the experimental group, oroantral communication closure was performed using a resorbable heterogeneous collagen membrane (Creos Xenoprotect, Nobel Biocare, Herzogenrath, Germany). After confirmation of the communication and measurement of its diameter, the extraction socket was carefully debrided, and all granulation tissue was removed. Sharp bony margins were smoothed using a carbide bur under copious sterile saline irrigation to prevent membrane perforation and ensure adaptation. A limited mucoperiosteal elevation was performed only to the extent necessary to allow submucosal placement of the membrane without tension. No vertical releasing incisions were made. The collagen membrane was trimmed extraorally to slightly exceed the defect margins and inserted in a submucosal position to completely cover the oroantral opening. Care was taken to ensure intimate adaptation to the surrounding bone and soft tissue. Stabilization was achieved using a horizontal mattress suture. The needle was passed from the buccal mucosa to the palatal mucosa and back to the buccal side, creating crossed sutures over the defect to secure the membrane in a stable position. Tension-free closure was verified clinically. The membrane was not exposed intentionally and was allowed to integrate and resorb spontaneously. Postoperative management, including antibiotic therapy, sinus precautions, analgesic protocol, and suture removal at 14 days, was identical to that in the control group to minimize confounding.

### 2.7. Surgical Procedure: Control Group

In the control group, a plastic surgery of the oral and sinus connection was performed using the Rehrman Buccal Advancement Flap: it is the most common method of closing oroantral communications (OACs) or fistulas (OAFs) [18]. In each patient across both groups, postoperative oral antibiotic therapy was administered in the form of penicillin with clavulanic acid (most commonly Augmentin 1000 mg, one tablet every 12 h). For patients allergic to penicillin, clindamycin was used at a dose of 600 mg every 12 h. Sutures were removed 14 days after the procedure. In the control group, oroantral communication closure was performed using the classical Rehrmann buccal advancement flap technique. Following curettage of the extraction socket and smoothing of sharp bony margins, a trapezoidal full-thickness mucoperiosteal flap was elevated from the buccal aspect using two vertical releasing incisions. The flap was carefully mobilized by periosteal releasing incisions to allow tension-free coronal advancement. Primary closure of the defect was achieved by advancing the flap over the communication and securing it with interrupted sutures, ensuring complete coverage of the socket opening. Postoperative management, including antibiotic therapy and suture removal at 14 days, was standardized across both groups.

### 2.8. Clinical and Radiological Assessment

During the first visit, patients underwent a thorough two-stage examination. The first stage involved a comprehensive medical and dental history, which was conducted using a specially designed questionnaire. For individuals qualified for tooth extraction, a clinical examination was performed, including measurement of the depth of the oral vestibule, the width of the keratinised gingiva using a standard WHO periodontal probe (Verdent, Łódź, Poland) ending with a ball tip divided every 1 mm, as well as radiological assessments, specifically panoramic X-ray, periapical X-ray, or cone beam computed tomography (CBCT), carried out to evaluate the condition of the mucosa of the maxillary sinus and the position of the tooth roots relative to the maxillary sinus cavity. On the first day after the procedure, volumetric tomography with a narrow imaging field of 5 × 5 was also performed. Computed tomography (CT) and CBCT scans are the gold standard modality of radiological assessment to rule out the presence of maxillary sinusitis [7]. Preoperative radiological assessment was performed according to routine clinical indications and the ALARA principle. Panoramic or periapical radiographs were used for standard extraction planning, as routine CBCT for all patients would involve a higher radiation dose and substantially greater cost. Cone-beam computed tomography was therefore reserved for cases in which detailed evaluation of sinus proximity or mucosal status was clinically required. In the present study, limited-field CBCT was obtained after confirmation of oroantral communication (day 0) and repeated at 90 days for standardized linear measurements and relative density assessment.

### 2.9. Postoperative Care and Follow-Up

Then, a surgical procedure was performed, along with instructions on oral hygiene and an explanation of post-operative care. The status of microbiota in the oral cavity may vary depending on the oral care products used, and the following can have a significant effect on the development of an inflammatory condition [8,9]. Detailed photographic documentation was also maintained. Each patient was committed to attending follow-up appointments before undergoing the examination. All patients were previously informed and agreed to attend postoperative follow up visit on the 1st, 7th and 14th day after the surgery. Each time, parameters such as wound healing, measurements of the depth and width of the keratinised gingiva, the effectiveness of the closure of the oroantral communication, and the pain scale according to the visual analogue scale (VAS) were assessed.

### 2.10. Pain Assessment

**Figure 1:**
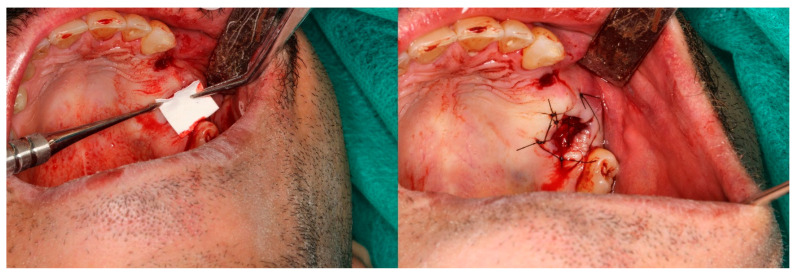
Closure of the oro-sinus connection using a heterogenous membrane.

**Figure 2:**
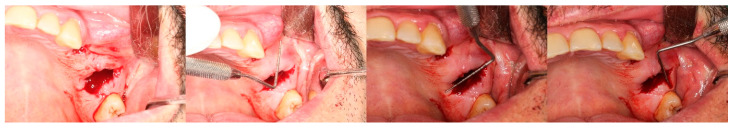
Measurements of the keratinised gingiva width, vestibular depth, and alveolar socket width on day 0.

**Figure 3:**
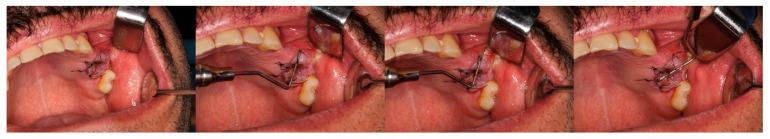
Measurement of the keratinised gingiva width, vestibular depth, and alveolar socket width on day 1.

**Figure 4:**
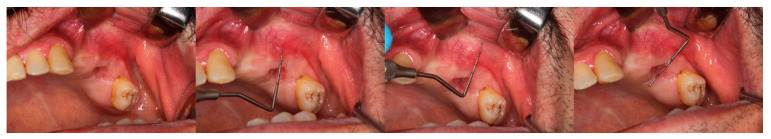
Measurement of the keratinised gingiva width, vestibular depth, and alveolar socket width on the 14th.

**Figure 5:**
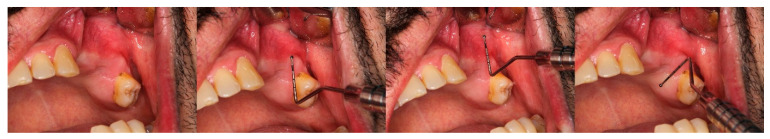
Measurements of the keratinised gingiva width, vestibular depth, and alveolar socket width on day 90.

The Visual Analogue Scale is a validated unidimensional tool for measuring acute postoperative dental pain intensity (Figure 1). This 100 mm horizontal line ranges from 0 (“no pain”) on the left to 10 (“worst pain imaginable”) on the right, with patients marking their current pain level; distance (mm) from the left anchor yields a continuous score from 0–100. Its high reliability, sensitivity to change, and ease of use make VAS the gold standard for postoperative oral surgery pain assessment, superior to verbal scales for detecting treatment differences. Accurate results require patients to understand endpoint anchors, with repeated measurements enabling evaluation of analgesic efficacy over time. Photographs were taken during each patient visit to assess the local condition (Figure 1, Figure 2, Figure 3, Figure 4 and Figure 5).

### 2.11. Final Follow-Up Examination

The final stage of the examination was carried out three months (90 days) after the procedure. Changes in all analyzed parameters were compared to assess the effectiveness of both methods. Additionally, a repeated CBCT scan was performed to evaluate the condition of the mucous membrane of the maxillary sinus and the reconstruction of bone tissue in the post-extraction defect.

### 2.12. Radiographic Measurements

Radiographic analysis consisted of measuring alveolar atrium dimensions, which were taken before the procedure, after 1, 14, and 90 days after surgery. The measurements were made using Romexis software (Version 6.4) provided by the Planmeca company. An assessment of the width of keratinised gingiva, the depth of the atrium, and the pain threshold was performed.

### 2.13. Linear Bone Measurements

The width between the buccal and palatal bony plates was measured at the alveolar crest, the bottom of the alveolus, and in the middle. The height was measured from the bottom of the alveolus to the top of the buccal and palatal bony plates, respectively.

### 2.14. Bone Density Assessment

Bone optical density in Hounsfield Units (HU) was measured in ImageJ software (version 1.53k, Wayne Rasband and contributors, National Institutes of Health, Kensington, MD, USA).

### 2.15. Region of Interest Analysis and Normalization

**Figure 6:**
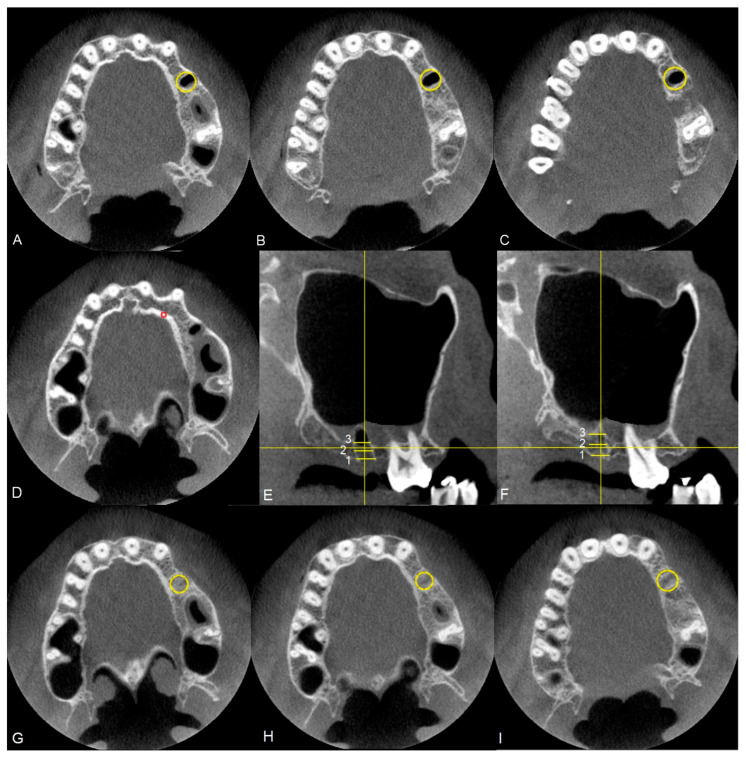
CBCT. Methodology for selecting cross-sections for density measurements in the transverse plane, where (**A**) measurement at the socket, (**B**) measurement in the center of the alveolus, (**C**) measurement at the level of the neck of the extracted tooth, (**D**) density measurement for normalizing the bone density of the respective volunteer, (**E**) schematic representation of measurement sites on the sagittal section on day 0, (**F**) schematic representation of measurement sites on the sagittal section after 90 days, (**G**) measurement at the socket performed after 90 days, (**H**) measurement in the center of the alveolus performed after 90 days, (**I**) measurement at the level of the neck of the extracted tooth performed after 90 days. The measurement of the reference value of bone tissue density after 90 days from the procedure was carried out in the same manner as the measurements immediately after tooth extraction.

Measurements of bone tissue density and surrounding tissues were performed on images obtained from CBCT scans, appropriately for patients treated using a membrane and the Rehrmann method, in accordance with the methodology shown in Figure 6. Each time, an area (ROI—region of interest) was marked where average densities in Hounsfield units were collected at three different heights of the alveolar process, specifically near the sinus, in the middle of the alveolus, and at the level of the neck of the extracted tooth. Density measurements were also performed to normalize the density of the participant’s bone tissue (Figure 6A–D). It was decided that the obtained tissue density measurements would be referenced to the density of compact bone tissue to reduce the influence of individual variability within the test group. The value was directly calculated as the ratio of the mean density at the site of tooth extraction to the mean density of compact bone tissue. All measurements were performed on cross-sectional slices, with layer selection based on simultaneous control of the position relative to sagittal sections (Figure 6E). Measurements were taken for each group immediately after tooth extraction (Figure 6A–E) and after 90 days of observation (Figure 6F–H). From the densities calculated at the three heights, the arithmetic mean was determined and included in the statistical analyses.

### 2.16. Postoperative Care

All patients in both treatment arms received intensified perioperative supervision and standardized postoperative care, which may have contributed to the low overall complication rate observed in this cohort. Specifically, patients were managed by an experienced surgeon, received uniform antibiotic prophylaxis, detailed oral hygiene and sinus precaution instructions (avoidance of nose blowing, sneezing with closed mouth, and negative intra-sinus pressure), and were scheduled for close follow-up visits on postoperative days 1, 7, and 14. Early clinical monitoring enabled prompt identification of minor wound issues and reinforcement of postoperative compliance. This level of structured surveillance and supportive care may have minimized adverse events such as flap dehiscence, sinus contamination, or secondary infection, and therefore the complication profile reported in this study may not fully reflect outcomes in less controlled routine practice settings.

### 2.17. Statistical Analysis

Statistical analyses were performed using STATISTICA software, version 13.0 (StatSoft, Tulsa, OK, USA). Continuous variables are presented as mean ± standard deviation (SD). Statistical significance was defined a priori as *p* < 0.05. The normality of data distributions was evaluated using the Shapiro–Wilk test, and homogeneity of variances was assessed with Levene’s test. Between-group comparisons were conducted using independent-samples *t*-tests when parametric assumptions were met; in cases of assumption violation, the Mann–Whitney U test was applied as a non-parametric alternative.

The primary outcome variable was defined a priori as the change in vestibular depth from baseline to 90 days, as this parameter reflects preservation of soft tissue architecture following oroantral communication closure. Secondary outcomes included changes in keratinized gingiva width, alveolar socket dimensions, postoperative pain intensity assessed using the Visual Analogue Scale, incidence of postoperative complications, and normalized radiographic bone density values derived from CBCT analysis. Because multiple secondary endpoints were evaluated, between-group comparisons were adjusted for multiplicity using the Bonferroni correction, and the adjusted significance threshold was applied accordingly. The primary outcome retained a two-sided alpha level of 0.05, whereas secondary outcomes were interpreted as supportive and exploratory findings. Continuous variables are presented as mean ± standard deviation, normality was assessed using the Shapiro–Wilk test, homogeneity of variance using Levene’s test, and appropriate parametric or non-parametric tests were applied as indicated.

## 3. Results

The efficacy of closure of an oral-sinus junction formed after extraction of a tooth in the lateral maxillary region using resorbable heterogeneous membranes compared with closure of the junction using the classic Rehrmann method was evaluated.

### 3.1. Assessment of the Depth of the Vestibule

**Figure 7:**
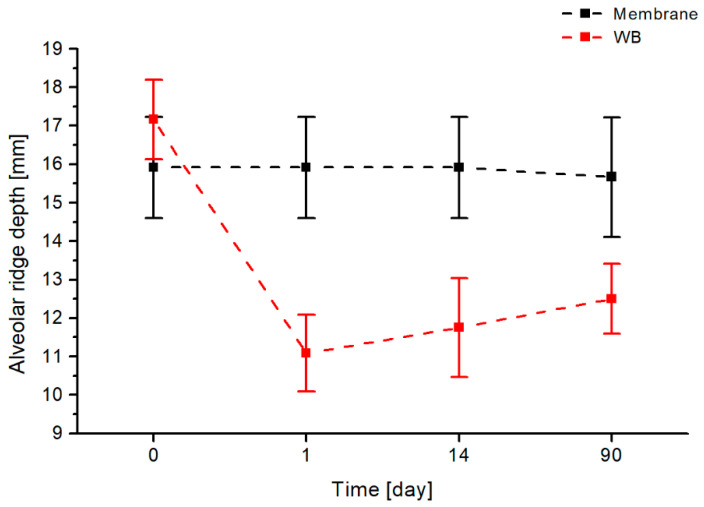
Comparison of changes in the depth of the vestibule over time for two evaluated methods of closing oroantral communications: the heterogenous membrane procedure (black) and the Rehrmann method (red).

In the first step, alveolar height dimensions were compared using the heterogeneous membrane and the Rehrmann method at a time interval of 90 days, and the results of this analysis are shown in Figure 7.

This graph clearly demonstrates that the use of the homogenous membrane maintains a constant vestibule depth value throughout the entire 90-day follow-up period after the procedure. With the classic Rehrmann method (without a membrane), a decrease in the vestibule depth value (from 17.17 mm to 11.08 mm) is observed as early as one day after the procedure. At 90 days after the procedure, this value does not increase (to 12.5 mm), but remains far from the values obtained with the heterogenous membrane.

**Figure 8:**
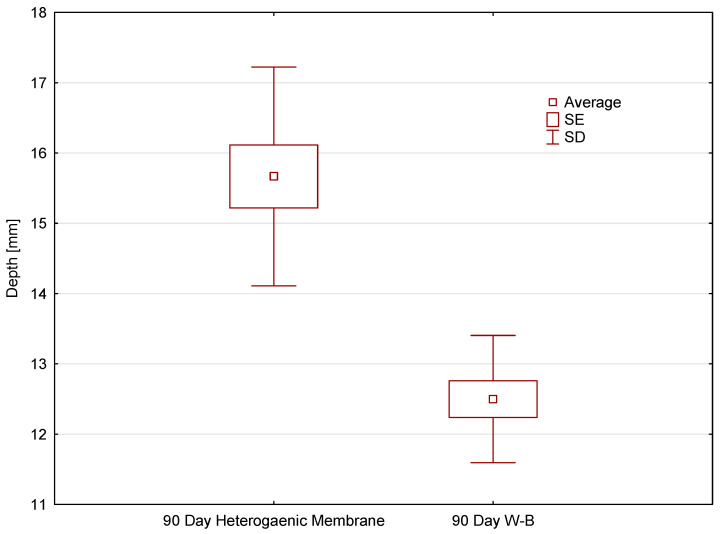
Average difference in vestibular depth after using the procedure with a heterogeneous membrane and the Rehrmann method, *p* < 0.05.

Statistical analysis of measurements taken before the procedure (0), one day after (1), on the 14th day, and 90 days after the procedure clearly indicates that the use of a collagen membrane significantly (*p* < 0.01) influences the preservation of the vestibular height and does not cause the development of pull syndrome, which could adversely affect subsequent implant-prosthetic treatment. On each day following the procedure, the differences in depth are statistically significant, and even 90 days after the procedure, the use of the membrane shows that the Rehrmann method maintains the reduction in the vestibule, as demonstrated in the chart below (*p* = 0.000004). The mean difference in the width of the vestibule after using the heterogeneous membrane procedure and the classic method is shown in Figure 8.

### 3.2. Assessment of the Keratinized Gingival Width

**Figure 9:**
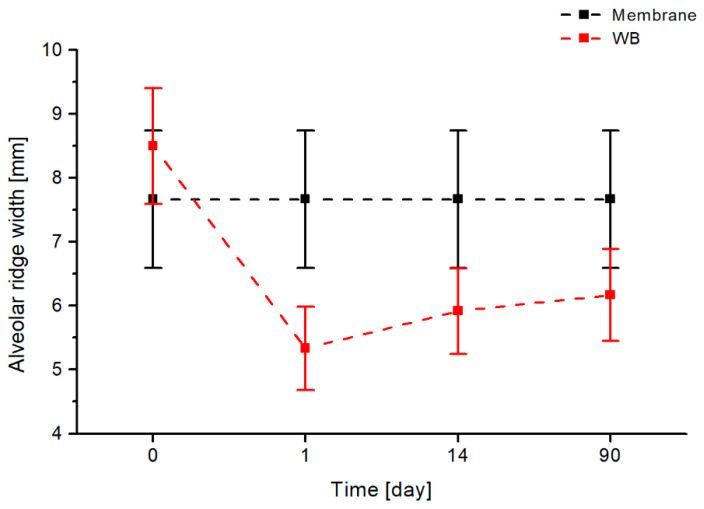
Comparison of changes in the width of keratinized gingiva over time for two evaluated methods of closing oroantral communications: the heterogenous membrane procedure (black) and the Rehrmann method (red).

In the next step, the width of the keratinised part of the gingiva was assessed and the results of this analysis are shown in Figure 9. Again, the use of a membrane keeps the width of the keratinized part of the gingiva constant (7.67 mm), whereas the classic method without a membrane results in a decrease in this value (from 8.5 mm to 5.33 mm) already on the first day of observation. In the following days, this value increases (to 5.92 mm after 14 days) and reaches a value of 6.17 mm 90 days after treatment. This final value differs from the keratinized gingival width value obtained with the membrane by 1.33 mm.

Statistical analysis of measurements taken before the procedure (0), one day after (1), on the 14th day, and 90 days after the procedure clearly indicates that the use of a collagen membrane allows the preservation of the anatomical width of the keratinized gum and enables the patient to undergo effective implant-prosthetic treatment in the future. On each day following the procedure, the differences in the width of the keratinized gum are statistically significant, and even three months after the procedure, the observed differences demonstrate a statistically significant advantage of the method using a collagen membrane.

### 3.3. Assessment of the Alveolar Socket Width

**Figure 10:**
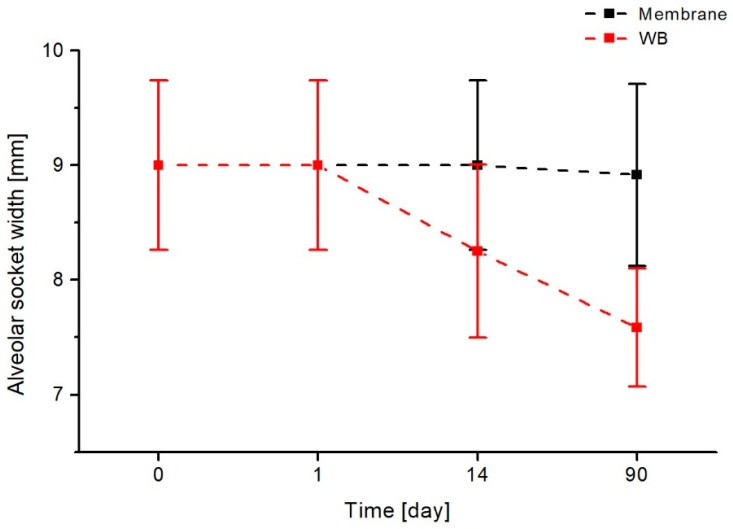
Comparison of changes in the width of the alveolar socket over time for two evaluated methods of closing oroantral communications: the heterogenous membrane procedure (black) and the Rehrmann method (red).

Subsequently, the width of the alveolar socket was assessed. The comparison of the alveolar socket width dimensioning using a heterogeneous membrane and the Rehrmann method over a 90-day interval is presented in Figure 10. This figure shows that, in both the membrane method and the Rehrmann method, the alveolar socket width remains at approximately 9 mm one day after the procedure. However, after 14 days, a noticeable decrease of 0.75 mm in alveolar socket width is observed with the Rehrmann method, and after 90 days, the reduction is 1.42 mm. At the same time, the use of a membrane maintains the alveolar socket width at a constant level throughout the entire observation period.

The analysis of the results of the conducted studies allowed the following conclusions to be drawn—in patients who underwent plastic surgery using the buccal advancement flap method, the alveolar width decreased.

### 3.4. Assessment of the Dynamics of Tissue Density Changes

**Figure 11:**
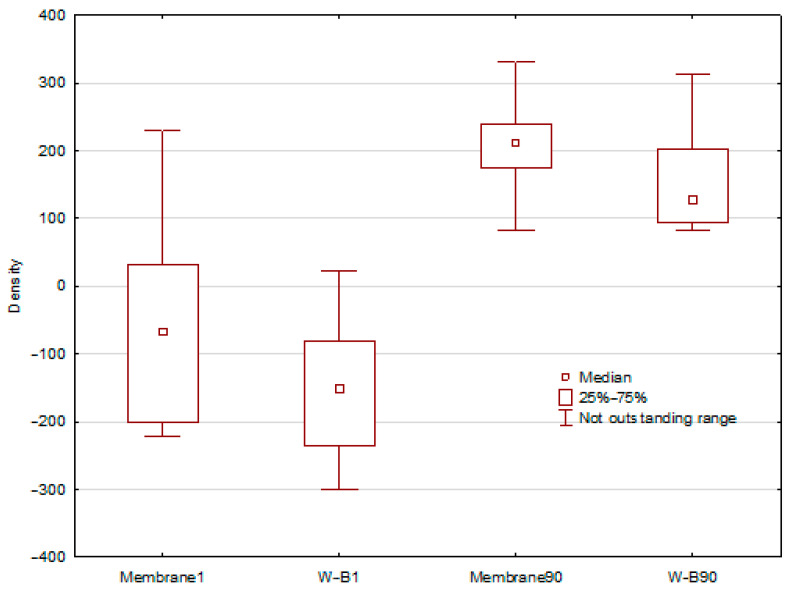
Median differences in tissue density in procedures using a heterogeneous membrane and the Rehrmann method on day 1 and day 90 after the performed procedure.

The final stage of the research was an assessment of the impact of applying a procedure using a heterogenous membrane and the buccal advancement flap method on the dynamics of tissue density changes at the site of the procedures. Figure 11 presents the differences in tissue density after 1 day and 90 days following the procedure. One day after tooth extraction, differences in density values between the membrane method and the Rehrmann method were noted, amounting to approximately 100 HU, whereas after 90 days, this difference decreased to about 50 HU. However, each time the use of the membrane resulted in a higher recorded tissue density value. Although normalized density values were consistently higher in the membrane group, these differences did not reach statistical significance. Given the limited sample size, radiographic density outcomes should be interpreted as exploratory and hypothesis-generating rather than definitive evidence of superior bone regeneration.

**Figure 12:**
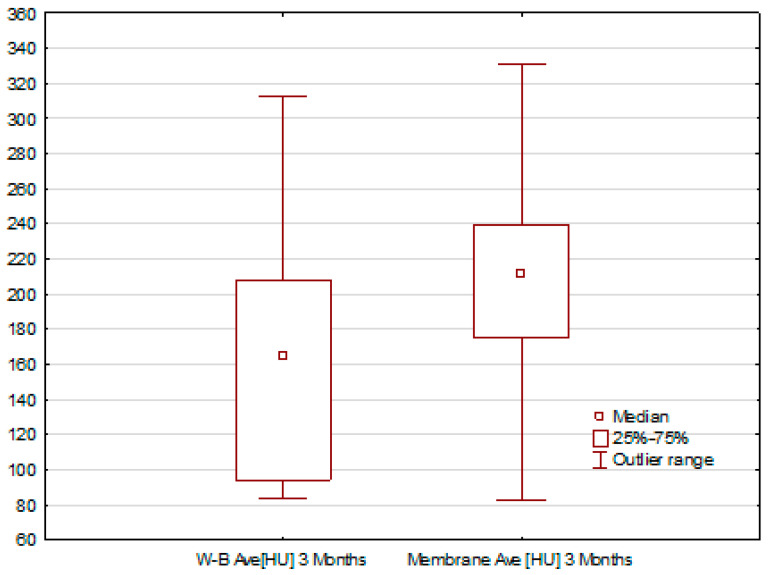
Normalized mean tissue density values at the site of the procedure, the heterogenous membrane and the Rehrmann method.

Furthermore, to better illustrate the differences in the density of reconstructed tissue between the two procedures, Figure 12 presents the medians of the normalized tissue density at the operation site 90 days after performing the respective procedures.

The obtained results and the presented charts (Figure 11 and Figure 12) indicate that the median bone density in the alveolar ridge, measured for the procedure using a membrane, is slightly higher than in the case of the Rehrmann procedure. However, these differences are not statistically significant. Normalized density values were consistently higher in the membrane group; however, these differences did not reach statistical significance. Therefore, the radiographic findings should be interpreted as preliminary trends in early mineralization rather than definitive evidence of superior bone regeneration. Nonetheless, expanding the research group and increasing the statistics may lead to more preliminary results. In the case of normalized means, tissue density values after 90 days from the procedure are, on average, 60% higher for the procedure using a membrane. However, these differences are still not statistically significant. Nevertheless, these results are consistent with expectations and correspond to the mean HU values.

### 3.5. Postoperative Complications

Analysis of postoperative complications revealed that in the test group, no cases of swelling, nosebleeds, or postoperative hematoma were observed. In contrast, in the control group, such cases were reported. No patient exhibited wound dehiscence, signs of re-anastomosis of the oral and sinus cavities, or oronasal fistula, nor suppuration of the postoperative haematoma or sinusitis.

### 3.6. VAS Pain Scale

**Figure 13:**
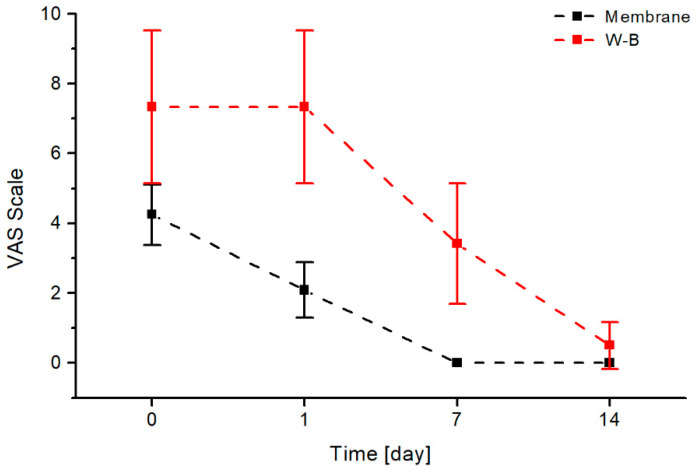
Comparison of changes in the VAS pain scale over time for two evaluated methods of closing oroantral communications: the heterogenous membrane procedure (black) and the Rehrmann method (red).

In the next step of the research, the impact of using a heterogeneous membrane procedure and the Rehrmann method on patients’ pain sensations was compared, with assessments conducted using the VAS pain scale. Figure 13 presents a comparison of the intensity of pain symptoms using the visual analogue scale (VAS), applied with a heterogeneous membrane over a 14-day interval.

The results indicate that patients who underwent plastic surgery using the buccal advancement flap experienced significantly stronger pain symptoms. A comparative analysis of the pain threshold for both treatment methods revealed that the perception of pain when using a collagen membrane on the first day after the procedure decreased twofold and continued to decrease in subsequent days. The adjustment of the pain threshold value determined by the patients in both methods occurs only after 14 days, when patients rarely report any pain complaints. The values obtained to describe pain symptoms for the method using membranes on each measurement day are statistically significantly lower (*p* < 0.01) than those for the Rehrmann method. The obtained results unequivocally confirm the effectiveness of membrane application in maintaining patient comfort and quality of life following surgery for the oroantral communication.

## 4. Discussion

### 4.1. Clinical and Biological Advantages of Heterogeneous Collagen Membranes in Acute Oroantral Communication Closure

The present study demonstrates that resorbable heterogeneous collagen membranes provide superior clinical outcomes compared to the traditional (Rehrmann buccal advancement flap) technique for acute OAC closure, particularly in preserving soft tissue architecture and reducing postoperative morbidity. These findings align with emerging evidence favoring minimally invasive biomaterial-based approaches over conventional flap techniques.

There are few articles where very little data for the usage of heterogenous membranes as an independent grafting material in the treatment of acute OAC. The primary aim of the work was to demonstrate the effectiveness of closing the oroantral communications using heterogenous membranes. The grafts used in this study are described as bone substitutes. This graft is volume-stable, biodegradable and osteoconductive. Under in vivo conditions, collagen membranes undergo resorption and exhibit good biocompatibility. They are non-toxic and do not induce any immunological or thermal interactions with the bone. Due to the minimally invasive nature of the procedure, the absence of incision and preparation of the mucoperiosteal flap, this method allows for the preservation of the appropriate height and thickness of the keratinised gingiva. It is an essential element for the long-term success of prosthetic treatment using fixed prostheses, as well as in justified cases of implant-prosthetic treatment. Patients do not require a secondary surgical procedure, such as vestibuloplasty. The postoperative healing results were consistent across all patients in the test group.

Conversely, after the closure of the orosinusal connection using the buccal mucoperiosteal flap, authors like Muglali or Burić claim that the shallow vestibule does not undergo spontaneous reformation and remains shallow for life [12,13]. Other authors like Beck suggest that within 8 weeks, the depth of the vestibule partially increases, but never returns to the original state [14].

Another important element in assessing the effectiveness of this method is evaluating the height and width of the alveolar process at the operated site, which is crucial for determining the ‘bone volume’ of the alveolar process. The preservation of the walls constitutes an essential element of the bone scaffold foundation, in which the forming clot enclosed by a heterogeneous membrane on the dorsal side induces proper bone regeneration. Following this line of research, the next stage in assessing the structure of bone regeneration is the analysis of the bone structure within the post-extraction alveolus using optical density measurement of the bone in Hounsfield Units.

The final element, which is extremely important for the treated patients, is maintaining post-operative comfort and quality of life, which was analysed using the visual analogue scale (VAS). According to the researchers, pain symptoms accompany the post-operative period following the closure of the OAC with any surgical technique [12]. In the studies conducted on the group using heterogenous membranes, due to the lack of necessity for wide, trapezoidal incisions, patients experienced significantly less pain than those in the control group using the traditional method. Such minimal pain allows participants to continue working and to function normally in society.

In our study, it was found that VAS (post-operative pain) was significantly less in the experimental group than the control group at 24 h and on the 7th and 14th post-operative days. The *p* value was statistically significant in all observations (*p* < 0.01). Additionally, no serious complications such as massive post-operative swelling, increased tissue tension, elevated body temperature, or severe pain requiring high doses of non-steroidal anti-inflammatory drugs occurred in the test group.

Although collagen membranes may provide an osteoconductive environment and support clot stabilization, the present study was not sufficiently powered to demonstrate statistically significant differences in CBCT-derived density outcomes. Consequently, any apparent increase in normalized HU values should be regarded as exploratory and hypothesis-generating.

### 4.2. Results in the Context of Other Evidence

#### 4.2.1. Comparison with Buccal Advancement Flap Studies

The current study’s findings corroborate the well-documented complications of buccal advancement flaps reported in the literature [20,21,22,23,24]. Bereczki-Temistocle et al. demonstrated that among 72 patients treated with buccal advancement flaps, 25 (34.7%) experienced relapses, with all large defects (6–15 mm) showing treatment failure [25]. Similarly, Visscher et al. reported a 10.4% recurrence rate requiring secondary intervention after Rehrmann flap closure [26]. The present study’s observation of vestibular depth reduction from 17.17 mm to 11.08 mm at day 1 post-traditional procedure, persisting at 12.5 mm at 90 days, confirms the permanent anatomical distortion described by Muglali and others [25,27]. In contrast, the membrane group maintained constant vestibular depth (17 mm) throughout follow-up, supporting the minimally invasive advantage of biomaterial approaches.

#### 4.2.2. Collagen Membrane Success Rates

The 100% closure success rate achieved with Creos Xenoprotect membranes in this study aligns with recent case series by Passarelli et al., who reported 100% closure in 12 patients using heterologous cortico-cancellous grafts covered with resorbable collagen membranes, with radiographic bone regeneration at 6 months [28]. Lopez et al. similarly achieved 100% closure in 28 patients using collagen membranes with cortical lamina stabilization [29]. These outcomes compare favorably to the 90% success rates reported for both platelet-rich fibrin (PRF) and buccal advancement flaps in randomized trials by Hunger et al. and Bilginaylar, though direct comparison is limited by differences in defect size criteria and follow-up duration [30,31].

#### 4.2.3. Pain and Patient-Reported Outcomes

The twofold reduction in VAS pain scores on postoperative day 1 with membranes versus Rehrmann (*p* < 0.01) mirrors findings from PRF studies. Bilginaylar demonstrated significantly lower pain and swelling with PRF compared to buccal advancement flaps on days 1–3, while Hunger et al. reported significantly lower pain scores with PRF at 21-day follow-up (*p* = 0.005) [30,31]. The absence of swelling, hematoma, or nosebleeds in the membrane group versus their occurrence in the Rehrmann group parallels the reduced morbidity profile of minimally invasive techniques across multiple studies [31].

#### 4.2.4. Soft Tissue Preservation

The preservation of keratinized gingiva width (7.67 mm vs. 6.17 mm at 90 days, *p* < 0.01) and prevention of mucogingival border displacement represent critical advantages for future prosthetic rehabilitation. Hunger et al. specifically measured significantly lower displacement of the mucogingival border with PRF versus buccal advancement flaps, while Gedik and Erdem emphasized that traditional buccal flaps cause vestibular shortening and interference with prosthetic rehabilitation [30,32]. The present study’s quantitative documentation of these parameters at multiple time points provides robust evidence for membrane superiority in maintaining anatomical integrity.

#### 4.2.5. Bone Regeneration Findings

While the 60% higher normalized bone density (HU values) at 90 days with membranes did not reach statistical significance (likely due to sample size), the trend aligns with documented osteoconductive properties of collagen membranes. Passarelli et al. demonstrated radiographic bone reformation in all 12 cases at 6 months using heterologous grafts with collagen membranes, and other studies showed significantly superior bone height, volume, and density with concentrated growth factors versus suture repair in a trial of 30 patients [28,33]. The present study’s use of normalized HU measurements to account for individual variability represents a methodological strength, though longer follow-up (≥6 months) may be needed to detect statistically significant differences in bone regeneration.

#### 4.2.6. Defect Size Considerations

The inclusion of acute OACs ≤ 24 h post-extraction distinguishes this study from many reports addressing chronic fistulas. Hunger et al. noted that defect size significantly influenced PRF success, with larger defects requiring more PRF plugs [30]. Bereczki-Temistocle et al. found that all large defects (6–15 mm) treated with Rehrmann flaps failed [25]. While the present study did not stratify outcomes by defect size, the consistent success across all membrane cases suggests efficacy for the acute, small-to-moderate defects typical of immediate post-extraction OACs.

#### 4.2.7. Alternative Techniques

Medical literature increasingly supports alternatives to buccal advancement flaps. The buccal fat pad (BFP) flap achieved 100% success with zero relapses in Bereczki-Temistocle’s series of 49 patients, compared to 34.7% relapse with buccal flaps [25]. However, BFP requires surgical expertise and may cause aesthetic changes or nerve paresthesia [34,35]. Platelet-rich fibrin demonstrates comparable success (90%) with lower pain and no vestibular compromise, though it requires blood processing and may be less effective for larger defects [30,31]. The present study’s membrane technique offers similar minimally invasive advantages without requiring autologous tissue harvest or specialized preparation.

### 4.3. Limitations of This Study

This study has several limitations inherent to its clinical design. The primary constraint is the small sample size (*n* = 20 completers from 24 enrolled), which provided adequate power for detecting clinically significant differences in soft tissue parameters (vestibule depth, keratinized gingiva width) and pain but limited statistical power for radiographic bone density outcomes, where observed trends (25–60% higher normalized HU values) did not reach significance. The non-randomized group allocation introduces potential selection bias, as patients were not prospectively randomized, though strict inclusion/exclusion criteria minimized baseline imbalances. The 90-day follow-up captures early bone regeneration but precludes assessment of long-term outcomes such as complete alveolar remodeling, implant success rates, or fistula recurrence beyond 3 months. Single-surgeon performance across both groups ensured procedural consistency but limits generalizability to multi-center or varied operator experience settings. Additionally, reliance on two-dimensional CBCT measurements with manual ROI analysis introduces minor inter-observer variability despite normalization to cortical bone. Future multicenter randomized controlled trials with larger cohorts, extended follow-up (≥12 months), and automated density quantification are needed to confirm bone regeneration superiority and establish membrane use as first-line therapy for acute OACs.

Interpretation of bone density changes based on CBCT-derived Hounsfield Unit (HU) values requires caution. Unlike conventional multislice computed tomography, CBCT systems are not uniformly calibrated for absolute HU quantification, and voxel gray values may be affected by scatter radiation, beam hardening, field-of-view selection, and acquisition parameters [36,37,38]. Consequently, CBCT-based HU measurements may not provide fully reliable absolute bone density values and should be regarded as relative surrogate indicators rather than definitive quantitative densitometry [36,37,38,39]. In the present study, density values were normalized to adjacent cortical reference bone in an attempt to reduce inter-individual variability and improve internal comparability between time points. Nevertheless, these radiographic findings should be interpreted as exploratory trends in early mineralization dynamics, and future studies with larger cohorts and standardized quantitative imaging protocols are required to confirm potential differences in bone regeneration.

Because allocation was not randomized, the possibility of selection bias cannot be excluded, and unmeasured baseline differences between groups may have influenced outcomes. Therefore, these findings should be interpreted as preliminary and hypothesis-generating. Future multicenter randomized controlled trials with concealed allocation are necessary to confirm the comparative effectiveness of membrane-based closure.

The study was underpowered for radiographic outcomes, particularly CBCT-derived density measurements. A post hoc power analysis (α = 0.05, two-tailed) indicated that with 10 patients per group, the achieved power is low for detecting moderate effect sizes (Cohen’s d = 0.5), and adequate sensitivity is reached only for large effects (d ≥ 1.0). Therefore, the observed HU trends should be considered preliminary and require confirmation in larger, adequately powered trials with standardized quantitative imaging.

### 4.4. Clinical Implications and Future Directions

These findings establish resorbable collagen membranes (Creos Xenoprotect) as a clinically superior, minimally invasive alternative to the buccal flap for acute oroantral communication closure, offering preserved vestibule depth and keratinized gingiva width essential for future implant-prosthetic rehabilitation, significantly reduced postoperative pain (VAS halved on day 1), and zero complications versus control group events. This technique fulfills Visscher’s criteria for ideal OAC treatment through rapid execution, excellent tolerability, and optimal early soft tissue/bone healing, warranting immediate adoption as first-line therapy for defects identified within 24 h post-extraction in appropriately selected patients. Future multicenter RCTs should enroll larger cohorts (*n* ≥ 100/group), extend follow-up to 12+ months to assess long-term fistula recurrence and implant survival, incorporate automated CBCT volumetric analysis for definitive bone density superiority confirmation, and evaluate cost-effectiveness alongside broader OAC sizes and operator experience levels to refine treatment algorithms in oral surgery practice.

## 5. Conclusions

Within the limitations of this prospective non-randomized clinical study, resorbable heterogeneous collagen membranes demonstrated favorable clinical outcomes in the closure of acute oroantral communications compared with the buccal advancement flap. The membrane technique was associated with significantly better preservation of vestibular depth, keratinized gingiva width, and alveolar socket dimensions during the 90-day follow-up period, as well as lower postoperative pain scores. Radiographic assessment revealed higher normalized density values in the membrane group; however, these differences did not reach statistical significance and should be interpreted cautiously. These findings suggest that resorbable collagen membranes represent a clinically effective and minimally invasive option for the management of acute oroantral communications diagnosed within 24 h post-extraction. Further randomized studies with larger sample sizes and longer follow-up are required to confirm these results.

## Figures and Tables

**Table 1:** The selection criteria for this study.

Inclusion Criteria	Exclusion Criteria
Adults (≥18 years).	Head and neck neoplastic disease (diagnosis or treatment).
General health status permitting dental surgical procedures.	Pregnancy.
Qualification for single-tooth extraction.	Systemic diseases preventing outpatient surgical treatment.
Systemic diseases not contraindicating dental surgery.	Minors (<18 years).
Smokers and non-smokers.	Blood disorders.
Provision of informed written consent.	Anticoagulant therapy.
Acute OAC diagnosed within 24 h	Immunosuppressive therapy.
	Chronic inflammatory disease of the maxillary sinuses.
	Oroantral communication present for more than 24 h.
	History of multiple previous procedures.

## Data Availability

The original contributions presented in the study are included in the article, further inquiries can be directed to the corresponding author.
